# *N*′-(3-Chloro­benzyl­idene)-4-hy­droxy­benzohydrazide

**DOI:** 10.1107/S1600536812048325

**Published:** 2012-11-30

**Authors:** Syed Muhammad Saad, Itrat Fatima, Shahnaz Perveen, Khalid M. Khan, Sammer Yousuf

**Affiliations:** a H.E.J. Research Institute of Chemistry, International Center for Chemical and Biological Sciences, University of Karachi, Karachi 75270, Pakistan; b PCSIR Labortories Complex, Karachi, Shahrah-e-Dr. Salmuzzaman Siddiqui, Karachi 75280, Pakistan

## Abstract

The mol­ecule of the title compound, C_14_H_11_ClN_2_O_2_ adopts an *E* conformation of the azomethine double bond and the dihedral angle between the benzene rings is 38.96 (13)°. In the crystal, mol­ecules are linked by N—H⋯O and O—H⋯O (with the ketone O atom as acceptor) and C—H⋯O (with the hy­droxy O atom as acceptor) hydrogen bonds, forming a three-dimensional network.

## Related literature

For a related structure and background to the chemistry of the *N*-acyl­hydrazone unit, see: Taha *et al.* (2012 ▶). For a related structure, see: Hao (2009 ▶).
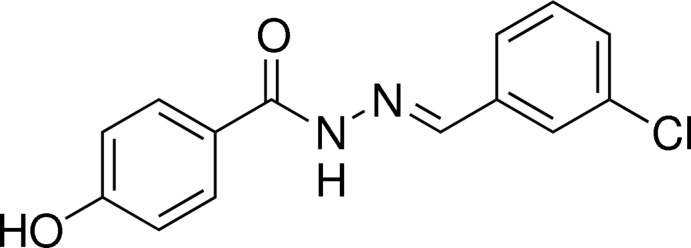


## Experimental

### 

#### Crystal data

C_14_H_11_ClN_2_O_2_
*M* *_r_* = 274.70
Orthorhombic, 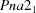

*a* = 9.0900 (8) Å
*b* = 9.9396 (9) Å
*c* = 13.8615 (12) Å
*V* = 1252.40 (19) Å^3^
*Z* = 4
Mo *K*α radiation
μ = 0.30 mm^−1^
*T* = 293 K
0.27 × 0.11 × 0.10 mm

#### Data collection

Bruker SMART APEX CCD diffractometer
Absorption correction: multi-scan (*SADABS*; Bruker, 2000 ▶) *T*_min_ = 0.923, *T*_max_ = 0.970
6999 measured reflections
2274 independent reflections
1980 reflections with *I* > 2σ(*I*)
*R*_int_ = 0.037

#### Refinement

*R*[*F* ^2^ > 2σ(*F* ^2^)] = 0.037
*wR*(*F* ^2^) = 0.081
*S* = 1.02
2274 reflections
180 parameters
1 restraint
H atoms treated by a mixture of independent and constrained refinement
Δρ_max_ = 0.21 e Å^−3^
Δρ_min_ = −0.20 e Å^−3^
Absolute structure: Flack (1983 ▶), 1060 Friedel pairs
Flack parameter: 0.12 (9)

### 

Data collection: *SMART* (Bruker, 2000 ▶); cell refinement: *SAINT* (Bruker, 2000 ▶); data reduction: *SAINT*; program(s) used to solve structure: *SHELXS97* (Sheldrick, 2008 ▶); program(s) used to refine structure: *SHELXL97* (Sheldrick, 2008 ▶); molecular graphics: *SHELXTL* (Sheldrick, 2008 ▶); software used to prepare material for publication: *SHELXL97*.

## Supplementary Material

Crystal structure: contains datablock(s) global, I. DOI: 10.1107/S1600536812048325/hb6997sup1.cif

Structure factors: contains datablock(s) I. DOI: 10.1107/S1600536812048325/hb6997Isup2.hkl

Supplementary material file. DOI: 10.1107/S1600536812048325/hb6997Isup3.cml

Additional supplementary materials:  crystallographic information; 3D view; checkCIF report

## Figures and Tables

**Table 1:** Hydrogen-bond geometry (Å, °)

*D*—H⋯*A*	*D*—H	H⋯*A*	*D*⋯*A*	*D*—H⋯*A*
N2—H2*A*⋯O1^i^	0.84 (2)	2.20 (2)	3.026 (3)	169 (3)
O2—H2*B*⋯O1^ii^	0.79 (3)	2.01 (3)	2.739 (3)	154 (3)
C4—H4*A*⋯O2^iii^	0.93	2.55	3.216 (3)	128

Symmetry codes: (i) 
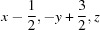
; (ii) 
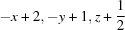
; (iii) 
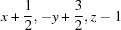
.

**Fig. 1.**
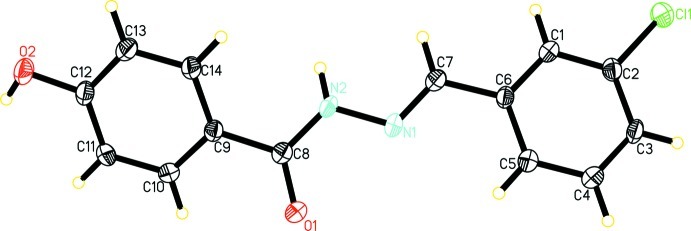
The molecular structure of (I) with displacement ellipsoids drawn at 30% probability level.

**Fig. 2.**
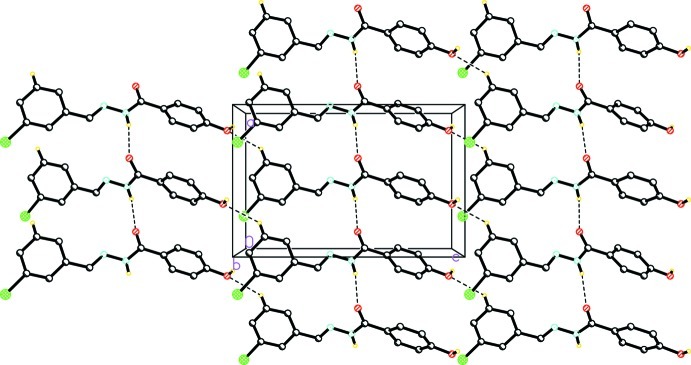
The crystal packing of the title compound I. Only hydrogen atoms involved in hydrogen bonding are shown.

## supplementary crystallographic information

### Comment

As part of our ongoing studies of the *N*-acylhydrazone moiety (Taha *et al.*, 2012), we now describe the structure of the title compound, which is similar to that of the previously published *N'*-(2-Chlorobenzylidene)-4-hydroxybenzohydrazide (Hao, 2009) with the difference that 2-chlorobenzne ring is replaced by 3-chlorophenyl ring (C1–C6). In the crystal, N2—H2A···O1, O2—H2B···O1,and C4—H4A···O2 hydrogen bonds link the moleucles into a three-dimensional-network (Table 2 and Fig. 2).

### Experimental

The title compound was synthesized by refluxing a mixture of 3-chlorobenzaldehyde (2 mmol, 0.23 ml), methanol (20 ml) and 3 drops of acetic acid. 4-hydroxybenzohydrazide (2 mmol, 0.304 g) was added into above mentioned mixture at ambient temperature and refluxed for 3 h with vigorous stirring. Progress of the reaction mixture was monitored by thin layer chromatography. After the completion of the reaction (TLC Analysis), the solvent of the reaction mixture was slowly evaporated at room temperature by keeping it in an open atmosphere in order to obtained colourless blocks (0.44 g, 80.3% yield).

### Refinement

H atoms on phenyl ring and methine carbon were positioned 0.93 Å (CH) and constrained to ride on their parent atoms with *U*_iso_(H)= 1.2*U*_eq_(CH). The H atoms on the nitrogen (N–H= 0.79 (3) Å) and oxygen (O–H= 0.89 (2) Å) atoms were located in difference fourier maps and refined isotropically.

### Crystal data

**Table:**

C_14_H_11_ClN_2_O_2_	*D*_x_ = 1.457 Mg m^−^^3^
*M**_r_* = 274.70	Mo *K*α radiation, λ = 0.71073 Å
Orthorhombic, *P**n**a*2_1_	Cell parameters from 1415 reflections
*a* = 9.0900 (8) Å	θ = 2.5–23.8°
*b* = 9.9396 (9) Å	µ = 0.30 mm^−^^1^
*c* = 13.8615 (12) Å	*T* = 293 K
*V* = 1252.40 (19) Å^3^	Block, colorles
*Z* = 4	0.27 × 0.11 × 0.10 mm
*F*(000) = 568	

### Data collection

**Table:**

Bruker SMART APEX CCD diffractometer	2274 independent reflections
Radiation source: fine-focus sealed tube	1980 reflections with *I* > 2σ(*I*)
Graphite monochromator	*R*_int_ = 0.037
ω scan	θ_max_ = 25.5°, θ_min_ = 2.5°
Absorption correction: multi-scan (*SADABS*; Bruker, 2000)	*h* = −11→11
*T*_min_ = 0.923, *T*_max_ = 0.970	*k* = −10→12
6999 measured reflections	*l* = −15→16

### Refinement

**Table:**

Refinement on *F*^2^	Secondary atom site location: difference Fourier map
Least-squares matrix: full	Hydrogen site location: inferred from neighbouring sites
*R*[*F*^2^ > 2σ(*F*^2^)] = 0.037	H atoms treated by a mixture of independent and constrained refinement
*wR*(*F*^2^) = 0.081	*w* = 1/[σ^2^(*F*_o_^2^) + (0.0383*P*)^2^] where *P* = (*F*_o_^2^ + 2*F*_c_^2^)/3
*S* = 1.02	(Δ/σ)_max_ < 0.001
2274 reflections	Δρ_max_ = 0.21 e Å^−^^3^
180 parameters	Δρ_min_ = −0.20 e Å^−^^3^
1 restraint	Absolute structure: Flack (1983), 1060 Friedel pairs
Primary atom site location: structure-invariant direct methods	Flack parameter: 0.12 (9)

### Special details

**Table:**

Geometry. All e.s.d.'s (except the e.s.d. in the dihedral angle between two l.s. planes) are estimated using the full covariance matrix. The cell e.s.d.'s are taken into account individually in the estimation of e.s.d.'s in distances, angles and torsion angles; correlations between e.s.d.'s in cell parameters are only used when they are defined by crystal symmetry. An approximate (isotropic) treatment of cell e.s.d.'s is used for estimating e.s.d.'s involving l.s. planes.
Refinement. Refinement of *F*^2^ against ALL reflections. The weighted *R*-factor *wR* and goodness of fit *S* are based on *F*^2^, conventional *R*-factors *R* are based on *F*, with *F* set to zero for negative *F*^2^. The threshold expression of *F*^2^ > σ(*F*^2^) is used only for calculating *R*-factors(gt) *etc*. and is not relevant to the choice of reflections for refinement. *R*-factors based on *F*^2^ are statistically about twice as large as those based on *F*, and *R*- factors based on ALL data will be even larger.

### Fractional atomic coordinates and isotropic or equivalent isotropic displacement parameters (Å^2^)

**Table:**

	*x*	*y*	*z*	*U*_iso_*/*U*_eq_	
Cl1	0.75369 (7)	0.98855 (8)	0.02100 (8)	0.0540 (2)	
O1	1.15017 (17)	0.62769 (17)	0.54009 (13)	0.0374 (4)	
O2	0.8459 (2)	0.6183 (2)	0.95100 (14)	0.0518 (6)	
H2B	0.874 (4)	0.549 (3)	0.971 (2)	0.059 (11)*	
N1	1.0036 (2)	0.7862 (2)	0.41778 (15)	0.0339 (5)	
N2	0.9609 (2)	0.7697 (2)	0.51292 (16)	0.0340 (5)	
H2A	0.878 (3)	0.799 (2)	0.529 (2)	0.032 (7)*	
C1	0.8540 (3)	0.9246 (3)	0.19819 (17)	0.0353 (6)	
H1B	0.7633	0.9546	0.2206	0.042*	
C2	0.8869 (3)	0.9307 (3)	0.10113 (19)	0.0359 (6)	
C3	1.0208 (3)	0.8895 (3)	0.06536 (19)	0.0391 (7)	
H3A	1.0407	0.8931	−0.0004	0.047*	
C4	1.1252 (3)	0.8426 (3)	0.1302 (2)	0.0437 (7)	
H4A	1.2175	0.8169	0.1078	0.052*	
C5	1.0943 (3)	0.8336 (3)	0.2265 (2)	0.0405 (7)	
H5A	1.1654	0.8008	0.2686	0.049*	
C6	0.9579 (3)	0.8729 (2)	0.26244 (17)	0.0317 (6)	
C7	0.9198 (3)	0.8545 (2)	0.36386 (18)	0.0354 (6)	
H7A	0.8344	0.8927	0.3887	0.043*	
C8	1.0415 (3)	0.6900 (2)	0.57049 (19)	0.0307 (6)	
C9	0.9925 (2)	0.6764 (2)	0.67131 (18)	0.0301 (6)	
C10	1.0357 (3)	0.5631 (3)	0.72305 (19)	0.0349 (6)	
H10A	1.0971	0.5003	0.6938	0.042*	
C11	0.9897 (3)	0.5416 (3)	0.81628 (18)	0.0357 (6)	
H11A	1.0196	0.4651	0.8496	0.043*	
C12	0.8986 (3)	0.6348 (3)	0.85996 (18)	0.0364 (6)	
C13	0.8576 (3)	0.7497 (3)	0.81142 (19)	0.0446 (7)	
H13A	0.7991	0.8137	0.8419	0.053*	
C14	0.9029 (3)	0.7698 (3)	0.71824 (18)	0.0400 (6)	
H14A	0.8735	0.8471	0.6857	0.048*	

### Atomic displacement parameters (Å^2^)

**Table:**

	*U*^11^	*U*^22^	*U*^33^	*U*^12^	*U*^13^	*U*^23^
Cl1	0.0529 (4)	0.0712 (5)	0.0378 (4)	0.0143 (4)	−0.0044 (4)	0.0075 (4)
O1	0.0349 (9)	0.0442 (10)	0.0330 (11)	0.0057 (8)	0.0062 (8)	−0.0020 (8)
O2	0.0712 (14)	0.0554 (15)	0.0289 (12)	0.0118 (11)	0.0139 (10)	0.0100 (11)
N1	0.0393 (12)	0.0384 (12)	0.0240 (12)	−0.0034 (10)	0.0052 (9)	0.0007 (9)
N2	0.0333 (11)	0.0452 (13)	0.0237 (12)	0.0008 (10)	0.0092 (11)	0.0022 (11)
C1	0.0346 (13)	0.0401 (15)	0.0311 (16)	0.0029 (12)	0.0072 (11)	0.0000 (11)
C2	0.0440 (15)	0.0345 (15)	0.0292 (15)	0.0000 (11)	−0.0006 (12)	0.0019 (11)
C3	0.0500 (16)	0.0418 (16)	0.0255 (15)	0.0020 (13)	0.0134 (12)	0.0053 (12)
C4	0.0408 (15)	0.0499 (18)	0.0403 (19)	0.0084 (13)	0.0128 (13)	0.0071 (14)
C5	0.0395 (14)	0.0500 (17)	0.0321 (16)	0.0065 (13)	0.0040 (12)	0.0058 (13)
C6	0.0386 (14)	0.0308 (14)	0.0257 (15)	−0.0007 (11)	0.0042 (11)	0.0033 (12)
C7	0.0364 (14)	0.0384 (16)	0.0315 (16)	0.0025 (12)	0.0065 (12)	−0.0004 (13)
C8	0.0299 (12)	0.0322 (14)	0.0299 (15)	−0.0051 (11)	−0.0001 (10)	−0.0018 (11)
C9	0.0295 (13)	0.0356 (15)	0.0252 (15)	−0.0020 (11)	−0.0003 (10)	−0.0020 (11)
C10	0.0355 (13)	0.0359 (15)	0.0332 (16)	0.0057 (12)	0.0009 (12)	−0.0021 (12)
C11	0.0401 (14)	0.0357 (16)	0.0313 (16)	0.0039 (12)	−0.0037 (12)	0.0073 (12)
C12	0.0380 (14)	0.0457 (17)	0.0253 (15)	−0.0034 (12)	0.0009 (12)	0.0010 (13)
C13	0.0578 (17)	0.0432 (16)	0.0327 (16)	0.0138 (13)	0.0094 (13)	−0.0026 (14)
C14	0.0520 (16)	0.0368 (15)	0.0311 (16)	0.0105 (13)	0.0046 (12)	0.0051 (12)

### Geometric parameters (Å, º)

**Table:**

Cl1—C2	1.741 (3)	C5—C6	1.392 (3)
O1—C8	1.240 (3)	C5—H5A	0.9300
O2—C12	1.359 (3)	C6—C7	1.460 (3)
O2—H2B	0.79 (3)	C7—H7A	0.9300
N1—C7	1.265 (3)	C8—C9	1.473 (3)
N1—N2	1.384 (3)	C9—C10	1.392 (3)
N2—C8	1.342 (3)	C9—C14	1.396 (3)
N2—H2A	0.84 (2)	C10—C11	1.375 (3)
C1—C2	1.380 (3)	C10—H10A	0.9300
C1—C6	1.396 (3)	C11—C12	1.382 (4)
C1—H1B	0.9300	C11—H11A	0.9300
C2—C3	1.376 (3)	C12—C13	1.377 (4)
C3—C4	1.388 (4)	C13—C14	1.370 (4)
C3—H3A	0.9300	C13—H13A	0.9300
C4—C5	1.368 (4)	C14—H14A	0.9300
C4—H4A	0.9300		
			
C12—O2—H2B	109 (2)	N1—C7—H7A	120.2
C7—N1—N2	117.2 (2)	C6—C7—H7A	120.2
C8—N2—N1	118.9 (2)	O1—C8—N2	121.9 (2)
C8—N2—H2A	122.6 (19)	O1—C8—C9	121.2 (2)
N1—N2—H2A	117.6 (19)	N2—C8—C9	117.0 (2)
C2—C1—C6	119.4 (2)	C10—C9—C14	117.6 (2)
C2—C1—H1B	120.3	C10—C9—C8	118.6 (2)
C6—C1—H1B	120.3	C14—C9—C8	123.9 (2)
C3—C2—C1	122.0 (2)	C11—C10—C9	121.6 (2)
C3—C2—Cl1	118.9 (2)	C11—C10—H10A	119.2
C1—C2—Cl1	119.1 (2)	C9—C10—H10A	119.2
C2—C3—C4	118.1 (2)	C10—C11—C12	119.3 (2)
C2—C3—H3A	120.9	C10—C11—H11A	120.3
C4—C3—H3A	120.9	C12—C11—H11A	120.3
C5—C4—C3	120.9 (2)	O2—C12—C13	117.2 (2)
C5—C4—H4A	119.6	O2—C12—C11	122.5 (2)
C3—C4—H4A	119.6	C13—C12—C11	120.3 (2)
C4—C5—C6	120.9 (3)	C14—C13—C12	120.0 (2)
C4—C5—H5A	119.5	C14—C13—H13A	120.0
C6—C5—H5A	119.5	C12—C13—H13A	120.0
C5—C6—C1	118.5 (2)	C13—C14—C9	121.1 (2)
C5—C6—C7	121.4 (2)	C13—C14—H14A	119.4
C1—C6—C7	120.0 (2)	C9—C14—H14A	119.4
N1—C7—C6	119.6 (2)		
			
C7—N1—N2—C8	175.3 (2)	N1—N2—C8—C9	179.5 (2)
C6—C1—C2—C3	−1.2 (4)	O1—C8—C9—C10	−21.1 (3)
C6—C1—C2—Cl1	177.28 (19)	N2—C8—C9—C10	157.1 (2)
C1—C2—C3—C4	−1.0 (4)	O1—C8—C9—C14	159.9 (2)
Cl1—C2—C3—C4	−179.4 (2)	N2—C8—C9—C14	−21.9 (3)
C2—C3—C4—C5	2.0 (4)	C14—C9—C10—C11	1.4 (4)
C3—C4—C5—C6	−0.8 (4)	C8—C9—C10—C11	−177.6 (2)
C4—C5—C6—C1	−1.4 (4)	C9—C10—C11—C12	−0.1 (4)
C4—C5—C6—C7	175.4 (3)	C10—C11—C12—O2	178.3 (2)
C2—C1—C6—C5	2.3 (4)	C10—C11—C12—C13	−1.7 (4)
C2—C1—C6—C7	−174.4 (2)	O2—C12—C13—C14	−177.8 (3)
N2—N1—C7—C6	−178.8 (2)	C11—C12—C13—C14	2.3 (4)
C5—C6—C7—N1	−9.2 (4)	C12—C13—C14—C9	−1.0 (4)
C1—C6—C7—N1	167.4 (2)	C10—C9—C14—C13	−0.8 (4)
N1—N2—C8—O1	−2.3 (3)	C8—C9—C14—C13	178.2 (3)

### Hydrogen-bond geometry (Å, º)

**Table:**

*D*—H···*A*	*D*—H	H···*A*	*D*···*A*	*D*—H···*A*
N2—H2*A*···O1^i^	0.84 (2)	2.20 (2)	3.026 (3)	169 (3)
O2—H2*B*···O1^ii^	0.79 (3)	2.01 (3)	2.739 (3)	154 (3)
C4—H4*A*···O2^iii^	0.93	2.55	3.216 (3)	128

Symmetry codes: (i) *x*−1/2, −*y*+3/2, *z*; (ii) −*x*+2, −*y*+1, *z*+1/2; (iii) *x*+1/2, −*y*+3/2, *z*−1.
